# Absence of Gut Microbiota Reduces Emotional Reactivity in Japanese Quails (*Coturnix japonica*)

**DOI:** 10.3389/fphys.2018.00603

**Published:** 2018-05-24

**Authors:** Narjis Kraimi, Ludovic Calandreau, Manon Biesse, Sylvie Rabot, Edouard Guitton, Philippe Velge, Christine Leterrier

**Affiliations:** ^1^UMR Physiologie de la Reproduction et des Comportements, National Institute of Agronomic Research, National Center of Scientific Research, University François Rabelais of Tours, Nouzilly, France; ^2^Micalis Institute, Institut National de la Recherche Agronomique, AgroParisTech, Université Paris-Saclay, Jouy-en-Josas, France; ^3^UE Plate-Forme d'Infectiologie Expérimentale, National Institute of Agronomic Research, Nouzilly, France; ^4^ISP, Institut National de la Recherche Agronomique, Université François Rabelais de Tours, UMR 1282, Nouzilly, France

**Keywords:** gut microbiota, microbiota-gut-brain axis, Japanese quail, germ-free, emotional reactivity

## Abstract

**Background:** Recent studies have demonstrated an effect of the gut microbiota on brain development and behavior leading to the concept of the microbiota-gut-brain axis. However, its effect on behavior in birds is unknown. The aim of the present study was to determine the effect of the absence of gut microbiota on emotional reactivity in birds by comparing germ-free (GF) quails to those colonized (COL) with gut microbiota.

**Material and Methods:** From hatching, the quails of both groups GF (*n* = 36) and COL (*n* = 36) were reared in sterile isolators. The COL quails were colonized at day 2 by introducing a pool of droppings from conventional adult females into the drinking water and feed. The quails were weighed individually on day 2, 6, and 14. From day 8, emotional reactivity was assessed in each group in the isolators through several behavioral tests.

**Results:** GF quails showed a considerable decrease in emotional reactivity demonstrated by spending less time in tonic immobility during the tonic immobility test (242 s ± 31 vs. 331 s ± 32, *p* ≤ 0.05), traveling a shorter distance (3,897 cm ± 242 vs. 4,827 cm ± 278, *p* ≤ 0.05) at a lower velocity (6.55 cm/s ± 0.4 vs. 8.1 cm/s ± 0.5, *p* ≤ 0.05) during the social separation test and spending more time near an object at the beginning of the novel object test (33.7 s ± 6.4 vs. 18.5 s ± 4.1, *p* ≤ 0.05). No difference in growth was found between the 2 groups.

**Conclusion:** For the first time, this study demonstrates that the absence of gut microbiota reduces emotional reactivity in Japanese quails in situations of fear and social perturbation without influence on growth.

## Introduction

Immediately after birth, the newborn gut is quickly and densely colonized by multiple microorganisms that constitute what is called the gut microbiota. It is now well-known that this gut microbiota is involved in many health processes such as digestion, metabolism or immune responses (O'Hara and Shanahan, 2006; Greer and O'Keefe, 2011; Kamada et al., 2013) and consequently is implicated in certain human diseases such as obesity or diabetes (Lê et al., 2013; Zhang et al., 2018). However, recent studies have also demonstrated an effect of these microorganisms on brain development (Bercik et al., 2011; Grenham et al., 2011; Collins et al., 2012; Sampson and Mazmanian, 2015) leading to the concept of the microbiota-gut-brain axis (Cryan and Dinan, 2012; Mayer et al., 2015). The mechanisms by which the gut microbiota communicate with the central nervous system are still poorly understood but could involve the bloodstream, immune responses via cytokines, hormone release by the endocrine system (Collins et al., 2012; Cryan and Dinan, 2012) or the vagal pathway (Bravo et al., 2011). Accumulating evidence suggests a strong influence of this microbiota-gut-brain axis on emotional behavior (Clarke et al., 2014; O'Mahony et al., 2015, for review). Indeed, in humans, the anxiolytic effect of probiotics (bacteria which have beneficial effects for the host when administered in sufficient quantity) and differences in gut microbiota composition between depressive and healthy patients have been demonstrated by several authors (Messaoudi et al., 2011; Naseribafrouei et al., 2014). In addition, many experiments using germ-free (living in the absence of detectable living microorganisms) rodents have indicated impaired anxiety-like behavior and hypothalamic-pituitary-adrenal (HPA) axis activity compared to specific pathogen–free rodents (Sudo et al., 2004; Diaz Heijtz et al., 2011; Crumeyrolle-Arias et al., 2014; Campos et al., 2016).

However, in birds, while the importance of gut microbiota on immunity, metabolism and growth performance has been well demonstrated for many years (Li et al., 2009; Brisbin et al., 2010; Lee et al., 2010), only a few studies have investigated its role in behavior. Toscano et al. (2010) showed that infection with *Salmonella enterica* led to a decrease in activity in chickens (reduced feeding and drinking) and a decrease in timidity (increased pecking at waxworms in a resource competition test). In addition, the administration of the probiotic *Bacillus amyloliquefaciens* helped to reduce agonistic behavior and distress calls in turkeys (Azeem, 2013). More recently, it has been shown that continuous supplementation from hatching with a probiotic (*Pediococcus acidilactici)* in Japanese quails caused a decrease in emotional reactivity in the tonic immobility test and improved memory (Parois et al., 2017). These results suggest that the gut microbiota is able to modify emotional behavior in poultry birds, as previously observed in rodents. Therefore, the aim of the present study was to determine the effect of the absence of gut microbiota on emotional reactivity in birds by comparing germ-free to colonized quails, an experiment never before conducted in birds. The Japanese quail (*Coturnix japonica*), a precocial bird, was chosen as a model in this study due to its capacities to live without its mother in early life, thus limiting the influence of maternal microbiota, and also because the effects of a probiotic on emotional behavior of this bird have already been demonstrated in previous work from our laboratory (Parois et al., 2017). It was recently reported that the host's genetic could influence the effects of the gut microbiota on behavior (Crumeyrolle-Arias et al., 2014). Hence in this study, we used a line of quails with a well-known genetic background. This line was genetically selected for its emotional reactivity level and more particularly a long duration of tonic immobility. Moreover, having no indication on the direction in which the absence of gut microbiota would influence emotional reactivity in quail, we decided to use a highly emotional line that should allow us to see a decrease or increase in emotional responses in our behavioral tests and in particular the tonic immobility test. Quails of the low emotional line with very short tonic immobility duration or even zero in some cases were less suitable because it would have been more difficult to observe a potential decrease in emotional reactivity.

## Materials and methods

Animal care procedures were carried out in accordance with the guidelines set by the European Communities Council Directive (86/609/EEC) and with French legislation on animal research (http://ethique.ipbs.fr/charteexpeanimale.pdf; 15 January 2011). Furthermore, the regional ethics committee approved the protocol (authorization number APAFIS#4191-2016022215316642).

### Eggs disinfection

Quail eggs were collected every 90 min from the farm of the avian experimental unit of INRA, Tours (UE PEAT, INRA, Nouzilly, France) so that they could be treated within 90 min of laying. They were immediately scrubbed with millliQ water and then disinfected with a mixture of potassium permanganate (10 g) and a solution of formalin + millliQ water for a minimum of 90 min. After this, the eggs were dipped in Divosan-plus (Diversey France SAS) 1.5% for 1 min and incubated for 14 days, in the facilities of the Plate-Forme d'Infectiologie Expérimentale: PFIE, UE-1277, INRA Centre Val de Loire, Nouzilly, France. After candling, the eggs underwent a third disinfection with a spray of Divosan 1.5% for 30 sec and were then rinsed with autoclaved water for 16.5 min and placed in sterile isolators for hatching.

### Animals and housing

**Figure 1:**
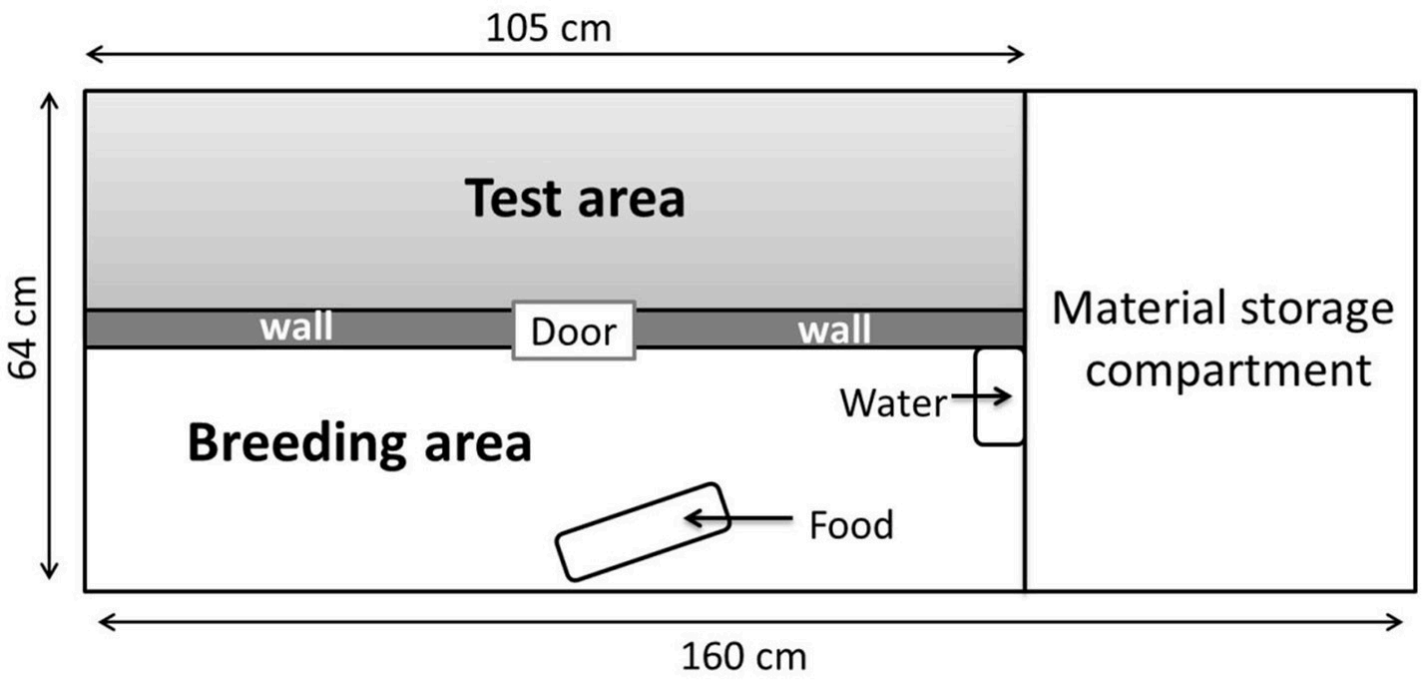
Different areas of the isolators (160 × 63 cm). The breeding area that contains food and water was separated from the test area by an opaque wall.

After the disinfection procedure, 72 Japanese quails hatched in germ-free conditions in sterile isolators, in the facilities of the PFIE, UE-1277. The quails originated from a line genetically selected for long tonic immobility duration and consequently a high level of emotional reactivity (Mills and Faure, 1991). Emotional reactivity is characterized by behavioral and physiological responses to a challenging situation (Boissy, 1995). On Day 2, chicks were transferred to six large sterile isolators of identical size (Figure 1) in the same room until they reached 14 days of age. The hatched quails were allocated to two groups: GF (germ-free, 22 males, 14 females) and COL (colonized, 19 males, 17 females) and housed in mixed-sex groups of 12 in isolators (3 isolators each for GF and COL groups). Surveillance for GF status was performed weekly through aerobic and anaerobic culture of freshly voided fecal samples (in resazurin thioglycolate, serum enriched BHI and blood agar and incubated at 35 and 25°C). Both GF and COL quails received the same autoclaved water and γ-irradiated (45kGy, Scientific Animal Food and Engineering, Augy, France) feed pellets for chicks (metabolizable energy: 12.2 MJ; Crude Protein: 204 g/kg). All quails were given free access to water and pellets. The temperature of the isolators was progressively reduced from 40 to 38°C the first days to 28°C at 14 days of age. The light cycle was 24 h of light from Day 0 to Day 4, and this was gradually reduced by 1 h of light per day until Day 14. An enrichment of the living environment of the chicks in the isolators was provided by placing previously sterilized new objects (glass or plastic balls) in the isolators on successive days and by wood shaving dust baths.

### Bacterial inoculation

Immediately on transfer to the isolators on Day 2, the chicks of the COL group were colonized by introducing a pool of droppings from 18 conventional adult females of the LTI line into the drinking water and feed. This means of colonization is similar to that occurring under natural conditions after hatching. The chicks were fasted for 90 min prior to addition of droppings to encourage feeding and promote a rapid colonization of their digestive tract. In each of the three isolators of the COL group, the colonization involved using 1 gram of droppings (+500 μl of sterile glycerol + cysteine) thawed and diluted in 10 ml of sterile physiological saline. 5 ml of this solution were added to the drinking water and 5 ml to the feed. The chicks of the GF group were fasted for the same period but did not receive the droppings supplement in their drinking water or feed. The colonization of microorganisms in the digestive tract of the COL group chicks was verified on Day 5 using the same protocol as for the surveillance of GF status.

### Behavioral tests

To maintain their bacterial status, the quails could not be removed from the isolators, the isolators were therefore divided into two equal parts using an opaque separating wall. One half was dedicated to rearing, containing the water and feed, while the other was specifically used for the behavioral tests (Figure 1).

Behavioral tests were always performed by the same experimenter with sterile gloves and equipment and were recorded with a camera fixed above each isolator.

#### Tonic immobility test

To evaluate the emotional reactivity of Japanese quails, the robust and standard tonic immobility test (Gallup, 1979; Jones, 1986; Mills and Faure, 1991) was performed at 8 days of age. Each quail was placed on its back in a U-shaped plastic cradle in the test half of the isolator and restrained for 10 s (with one hand on the sternum and the other lightly cupping the head of the bird). The experimenter remained silent and virtually motionless. If more than 10 s elapsed before the bird righted itself, the duration of tonic immobility was recorded. After five inductions without tonic immobility, 0 s was recorded. In contrast, if the quail failed to right itself after 10 min, the test was terminated and a maximum score of 600 s was recorded for tonic immobility duration. Two parameters were measured during the test: the number of induction attempts and the duration of tonic immobility. Quails with a low number of inductions and a long duration of tonic immobility were considered to be the most emotional. A third parameter calculated after the test was the tonic immobility index which gives higher weighting to tonic immobility induced easily and lower weighting to tonic immobility requiring many inductions [index= (6-number of inductions) × tonic immobility duration].

#### Social separation test

**Figure 2:**
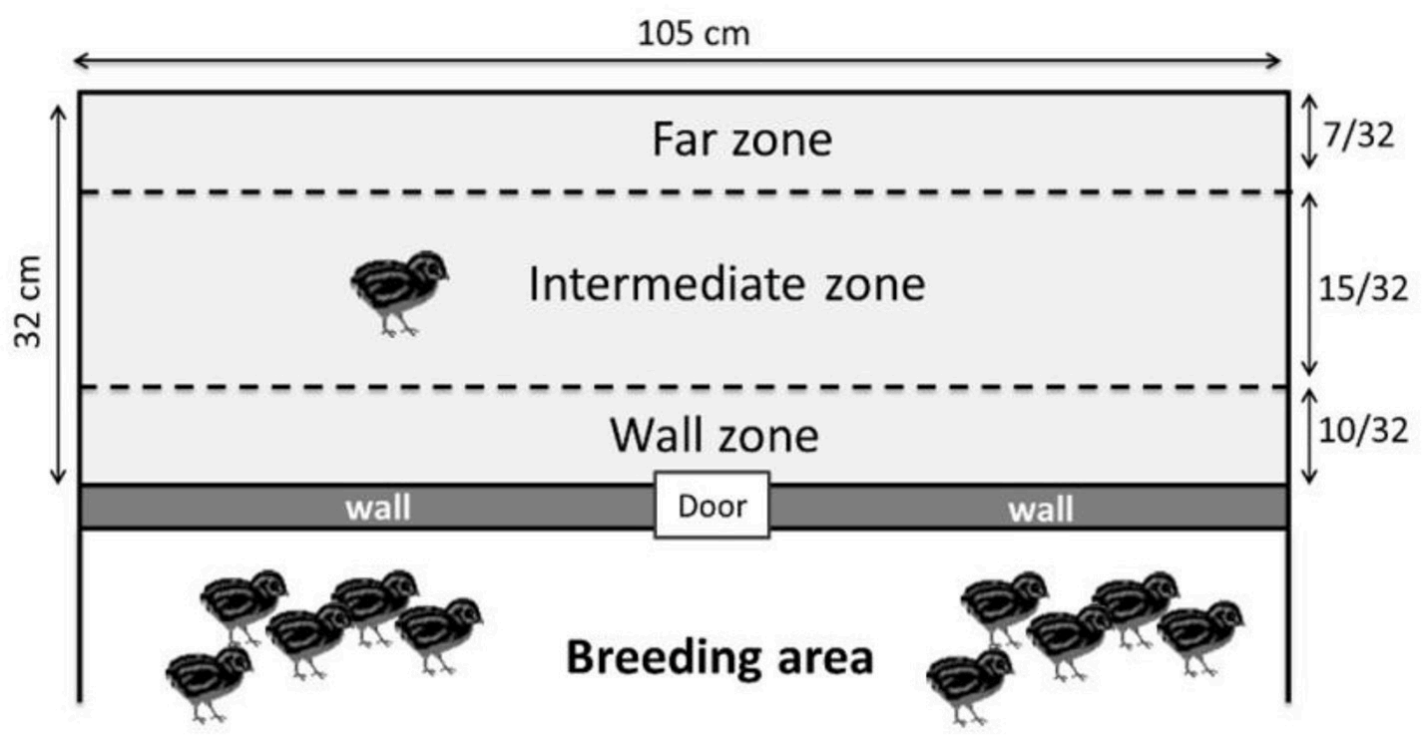
Different zones used for the social separation test (the wall zone, the intermediate zone and the far zone). The wall zone represents proximity with the other quails.

The day before this test, the quails were placed in groups of 3 individuals in the test compartment for 15 min to habituate them to this new environment. The social separation test was conducted at 10 days of age and measured the emotional reactivity of the quails during the stressful situation of being separated from its congeners. This separation cannot be called complete social isolation because in our isolator conditions, the quails could hear their congeners on the other side of the test wall. Each quail was individually placed in the test compartment of the isolator for 10 min. In this test, the total distance traveled in the whole test compartment and the velocity (distance traveled/time spent) of each quail were calculated using Ethovision XT software (Noldus Information Technology B.V., Wageningen, The Netherlands, VERSION 7.1). These results were used as a relevant indicator of the emotional reactivity because the emotional reactivity of the quail increases as it becomes more agitated in an attempt to find its congeners (Mills et al., 1993; Formanek et al., 2008; Schweitzer et al., 2010). The Ethovision XT software was also used to measure the number of entries and the time spent by the quails in each of the following zones of the test compartment (Figure 2): -An area close to the wall separating congeners: the “wall” zone-An area far from the wall separating congeners: the “far” zone-An intermediate zone between the two previous zones: the “intermediate” zone.

#### Novel object test

To test the emotional reactivity of quails in response to novelty, we recorded their behavior in the presence of a novel object (Miller et al., 2006). On Day 13, two quails (to limit the social isolation component) were simultaneously placed in the test compartment containing in the center a previously irradiated pink plastic worm (1 cm in diameter and 15 cm long) for 10 min.

The number of interactions with the object such as pecking it, considered as investigatory behavior in poultry (Hughes and Black, 1974) and moving the object were recorded with The Observer XT (version 12.5) software.

**Figure 3:**
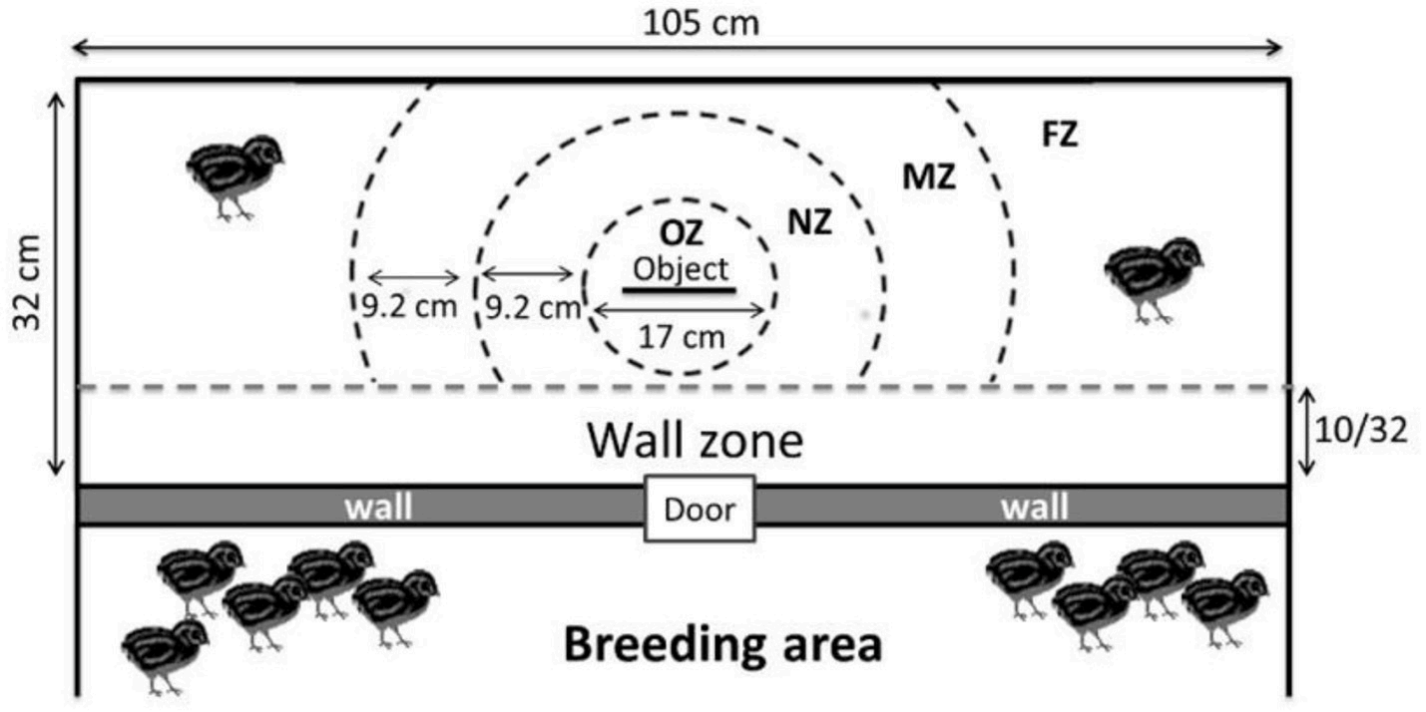
Different zones used for the novel object test (OZ, Object zone; NZ, Near object zone; MZ, Moderate object zone; FZ, Far object zone). The wall zone represents proximity with the other quails.

By defining zones around the object (Figure 3), the time spent close to or far from the object was also recorded.

### Growth

The quails were weighed individually in the isolators on Day 2, Day 6, and Day 14 with a system connected to an external balance.

### Statistics

Behavioral data were analyzed with R software using generalized linear mixed models (package “lme4”) with the treatment (GF or COL), the sex and the interaction between treatment and sex as fixed effects and with the order in which the quails have been tested as the random effect. We used generalized linear mixed models (GLMM) with different laws than a normal one when needed: a GLMM with Poisson errors was used to compare the numbers of inductions in tonic immobility test and the numbers of interactions with the object in the novel object test and a GLMM with Gamma errors was used for the total distance, the velocity and the time spent in the various zones during the social separation test and the novel object test. In the novel object test, where two quails were tested together, the pair number was used as the random effect. Growth data were first log-transformed and then tested using a generalized linear model with treatment as the main factor, age as the repetition factor and identification of each quail as the random factor. The results are presented as means ± SEM. The significance level was set at *p* ≤ 0.05 and 0.05 < *p* ≤ 0.10 was considered as a trend.

## Results

### Tonic immobility test

The sex effect and the interaction with the treatment were not significant for any of the following variables (*p* > 0.5, Table S1).

**Figure 4:**
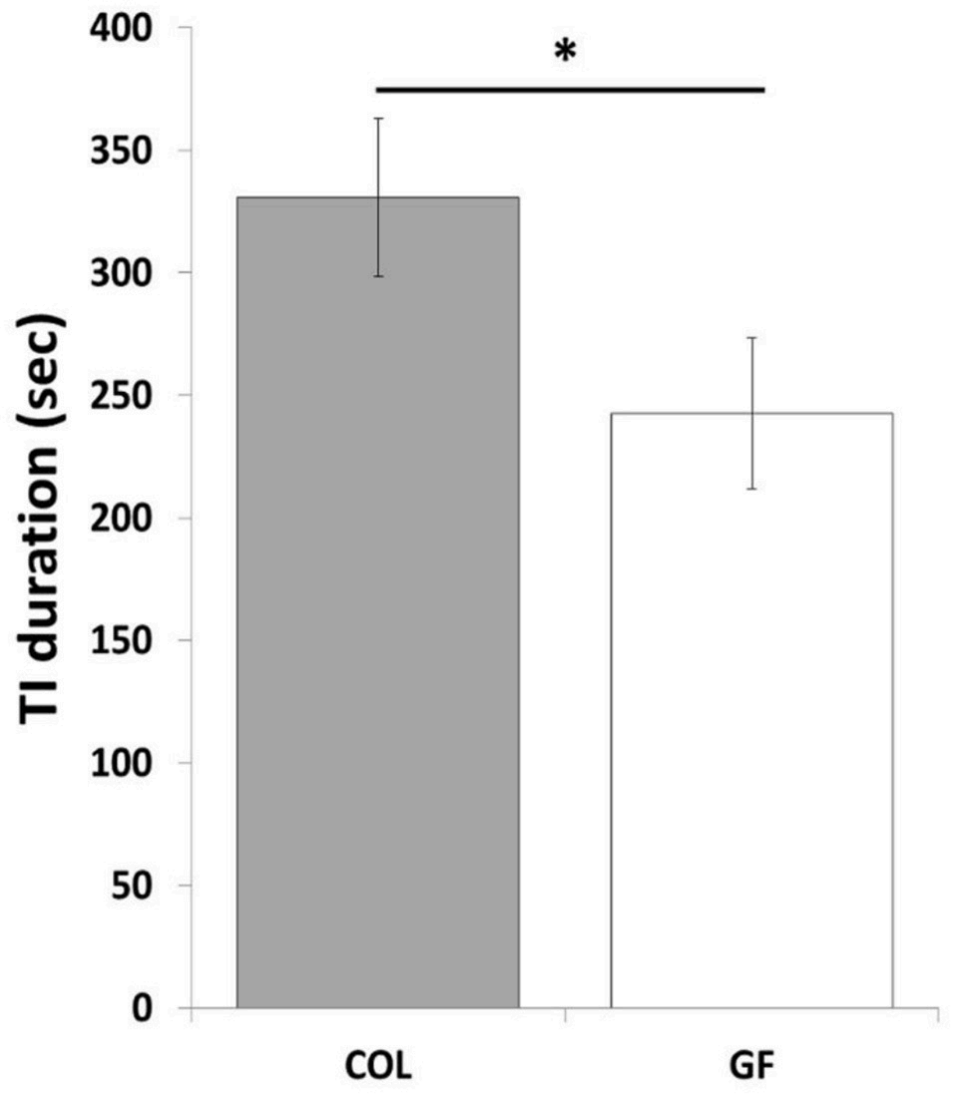
Tonic immobility duration of the COL (*n* = 36) and GF (*n* = 36) quails during the tonic immobility test. The results are expressed as mean values ± SEM. *p ≤ 0.05.

The number of inductions during the tonic immobility test did not differ between the GF and COL groups (χ^2^ = 0.70, *p* = 0.40). However, compared to the COL group, the GF group spent significantly less time in tonic immobility (Figure 4). Similarly, the tonic immobility index tended to show a lower value in quails of the GF group than for those of the COL group (1003.9 s ± 143.9 vs. 1397.3 s ± 154.5, χ^2^ = 3.43, *p* = 0.06).

### Social separation test

The sex effect and the interaction with the treatment were not significant for any of the following variables (*p* > 0.1, Table S1).

**Figure 5:**
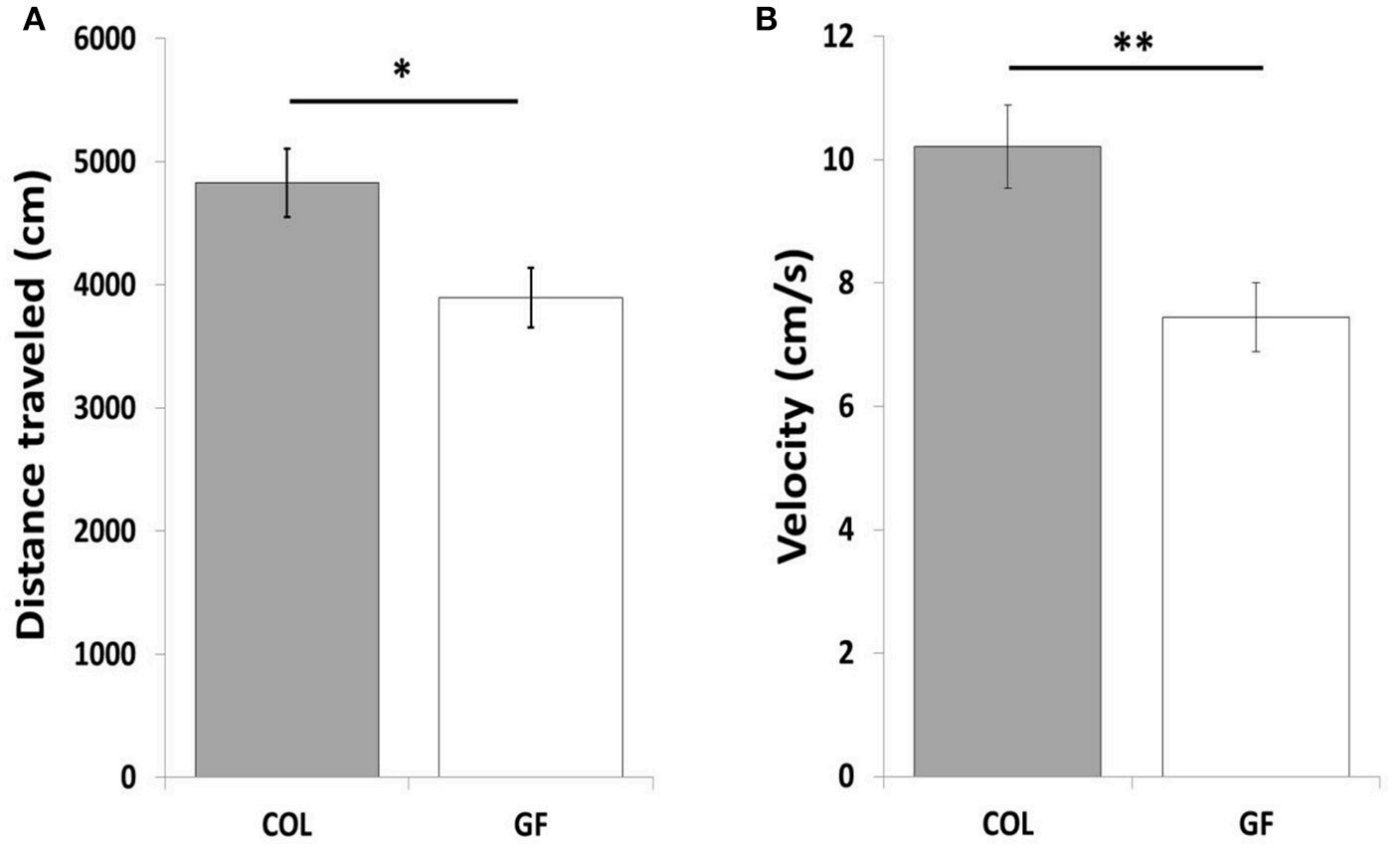
Total distance traveled **(A)** in the entire test area and movement velocity in the wall zone **(B)** of COL (*n* = 36) and GF (*n* = 36) quails during the social separation test. All results are expressed as mean values ± SEM. *p ≤ 0.05, **p ≤ 0.01.

The GF quails traveled a shorter total distance (Figure 5A) at a lower velocity (6.55 cm/s ± 0.4 vs. 8.1 cm/s ± 0.5, χ^2^ = 5.47, *p* ≤ 0.05) than the COL birds in the social separation test. Table 1 compares the number of entries and the time spent in the different zones of the test compartment between the two groups. The number of entries into the wall zone was significantly higher for COL than GF quails (χ^2^ = 6.42, *p* ≤ 0.01). This was accompanied by a similar trend for the intermediate and far zones (χ^2^ = 3.25, *p* = 0.07 and χ^2^ = 3.29, *p* = 0.07, respectively). No significant differences were observed for the time spent in the wall and far zones (χ^2^ = 2.15, *p* = 0.14 and χ^2^ = 1.28, *p* = 0.26, respectively). However, GF birds spent significantly less time in the intermediate zone than the COL birds (χ^2^ = 4.88, *p* ≤ 0.05). The distance traveled in the wall zone was similar for COL and GF quails (2615.2 cm ± 232.4 vs. 2384.2 cm ± 207, χ^2^ = 0.09, *p* = 0.75), but COL quails moved at a higher velocity (Figure 5B).

**Table 1:** Occupation of the test compartment zones by the COL (*n* = 36) and GF (*n* = 36) quails during the social separation test.

**Parameters**	**COL**	**GF**
Number of entries in wall zone	43.1 ± 4.6	29.1 ± 2.9^**^
Number of entries in intermediate zone	52.2 ± 5.7	38.5 ± 4.6^#^
Number of entries in far zone	16.8 ± 2.6	11.1 ± 1.7^#^
Time spent in wall zone (sec)	258.0 ± 23.2	325.0 ± 21.0
Time spent in intermediate zone (sec)	188.7 ± 28.4	122.5 ± 13.4^*^
Time spent in far zone (sec)	125.6 ± 18.9	92.5 ± 18.6

*All data in the table are expressed as mean values ± SEM*.

* *p ≤ 0.05*;

** *p ≤ 0.01*;

# *p ≤ 0.10*.

### Novel object test

The sex effect and the interaction with the treatment were not significant for any of the following variables (*p* > 0.2, Table S1).

**Figure 6:**
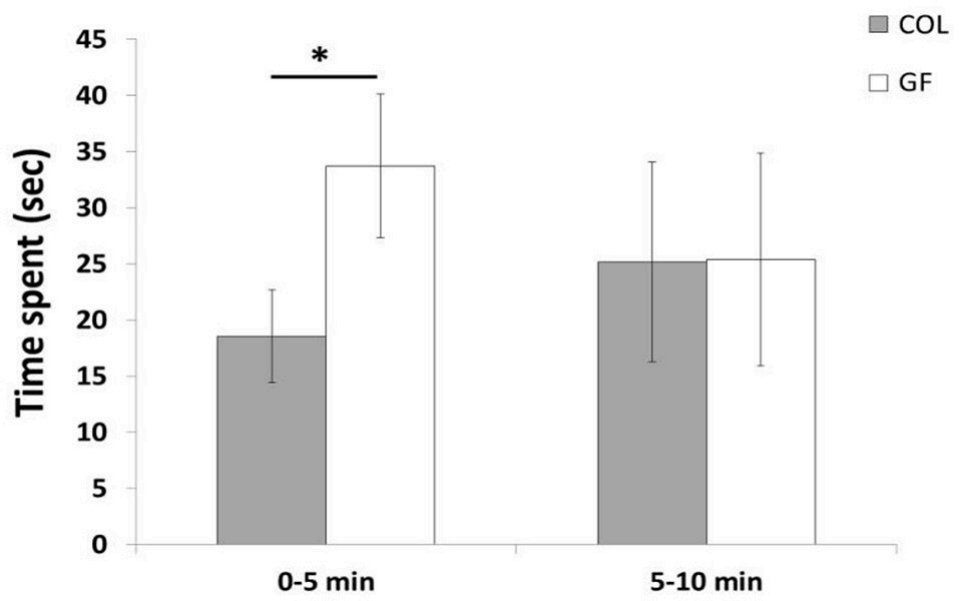
Time spent in the near object zone during the first 5 min and the last 5 min of the novel object test in the COL group (*n* = 36) and the GF group (*n* = 36). The results are expressed as mean values ± SEM. *p ≤ 0.05.

The number of interactions with the object such as pecking and moving it did not differ between the COL and GF groups (χ^2^ = 0.03, *p* = 0.84 and χ^2^ = 0.01, *p* = 0.97, respectively). Furthermore, during this test, only 17 of the 72 quails interacted with the object (8 GF and 9 COL). No significant difference between the COL and GF groups was found for the time spent in the wall zone, far object zone, moderate object zone, near object zone and object zone (190.7 s ± 31.3 vs. 267.7 s ± 33.8, χ^2^ = 1.18, *p* = 0.28, 260.9 s ± 30.8 vs. 191.5 s ± 29.6, χ^2^ = 1.33, *p* = 0.25, 94.3 s ± 21.6 vs. 73.2 s ± 10.6, χ^2^ = 0.09, *p* = 0.77, 43.7 s ± 10.2 vs. 59.1 s ± 13.3, χ^2^ = 0.76, *p* = 0.38, 10.3 s ± 5.5 vs. 8.4 s ± 3.7, χ^2^ = 0.52, *p* = 0.46, respectively). However, when examining the behavior during the first and second half of the test, we observed that GF quails spent significantly more time in the near object zone than the COL group in the first 5 min but during the last 5 min of the test, this difference disappeared (Figure 6). No significant difference was revealed between our two groups for the other zones for either of these time periods.

### Growth

The weight of the quails increased with age (*p* < 0.0001) but was not significantly different between the GF and COL groups [*F*_(1, 214)_ = 0.756, *p* = 0.39].

## Discussion

### Effects on emotional reactivity

The present work highlights a considerable decrease in the emotional reactivity of GF compared to COL quails in the tonic immobility test. Tonic immobility is a widely used test for assessing fear in poultry birds and it is a behavioral response which is positively related to high emotional reactivity (Gallup, 1979; Jones, 1986). It is also with this test that quails used in our study are selected for their high level of emotional reactivity. This genetic selection has been robust for many generations and it has been shown that the tonic immobility duration of these selected quails is also associated with high emotional reactivity responses in other tests such as open-field and hole-in-the-wall box tests (Mills and Faure, 1991) and related to autonomic responses (Valance et al., 2007). It is therefore all the more noteworthy to observe that this selected behavioral trait has been modified by the absence of gut microbiota. Indeed, GF quails spent less time in tonic immobility than COL quails. The tonic immobility index (which gives more weight to easily induced tonic immobility and lower weight to tonic immobility requiring many inductions) tended to confirm this result by tending to be greater in the GF than COL quails. In addition, this index for COL quails was equivalent to the tonic immobility index of quails of this line raised under normal conditions outside isolators during selection tests (Faure et al., 2006). This proves that the special conditions of the isolator rearing did not change the behavior of these COL quails selected for their high level of emotional reactivity. Moreover, it also suggests that the prenatal experience of germ-free chicks did not alter their reactivity, probably because they were produced by conventional mothers and the maternal environment was not affected by germ-free conditions.

During the social separation test, GF quails were also less emotional, traveling a shorter total distance than COL quails. It is therefore not surprising that the number of entries into the different zones of the test compartment was higher in COL quails. Furthermore, the distance traveled by the COL quails cannot be interpreted as greater exploration of the test area because the movement velocity was also significantly higher than for GF quails, a result which is characteristic of actively searching for congeners during a social isolation test (Mills et al., 1993; Formanek et al., 2008). Moreover, when looking more precisely at the activity of GF quails in the wall zone close to their congeners, it can be noted that they traveled a similar distance than COL quails but the movement velocity indicates that COL quails moved faster in this wall zone. This activity is very similar to pacing, a behavior commonly observed in response to social separation in Japanese quail (Schweitzer et al., 2010). In our case, it thus appears that GF quails were less emotional in this stressful situation.

In the novel object test, no significant difference was found between COL and GF quails over the entire test. Overall, they expressed very few interactions with the object, and spent little time in the zones close to the object. These findings that illustrate an avoidance of novelty are consistent with results in the literature (Richard et al., 2008). The quails of both groups seemed afraid of this novel object, but GF quails expressed a lower emotional reactivity at the beginning of the test by spending more time in the zone near the object than COL quails. Nevertheless, we did not find this difference during the last part of the test. This could be explained by the fact that even though we tried to suppress the social isolation component by introducing the quails in pairs, after a while, the social valence represented by the congeners just the other side of the wall was probably stronger than that of the object.

Overall, these results suggest that GF quails had lower emotional responses in situations that induced fear and social perturbation. Several studies in rodents have found similar results to ours. Germ-free mice showed greater exploration of an open-field and spent more time in the light part during a light-dark box test and more time in the open arms of the elevated plus maze test (Diaz Heijtz et al., 2011; Neufeld et al., 2011; Clarke et al., 2012; Arentsen et al., 2015). When the gut microbiota is not completely eliminated it can be modified by antibiotic administration and the use of antibiotics demonstrated anxiolytic effects in mice during the light-dark box test (Collins and Bercik, 2009). However, it is important to note that in some studies the absence of gut microbiota revealed no change in anxiety-like behavior (Gareau et al., 2011) and in other cases results opposite to ours. Indeed, other teams have observed an increase in the anxiety-like behavior responses of germ-free rodents in the marble-burying test and the open-field test (Nishino et al., 2013; Crumeyrolle-Arias et al., 2014).

Some authors have suggested that this lack of consensus between results could be explained by the different genetic strains of mice used between GF experiments, and that gut microbiota would buffer the strain effect, i.e., reducing emotional reactivity in anxious strains and enhancing it in strains with low anxiety (Crumeyrolle-Arias et al., 2014). In the present case, we used a genetic line selected for a high level of emotional reactivity using the tonic immobility test and other fear tests, consequently germ-free quails should thus be expected to be more emotional than colonized quails according to this hypothesis. However, we observed the opposite result and this could be due to the animal model chosen and the differences in the maternal environment (germ-free conditions in germ-free rodents vs. conventional conditions in germ-free quails). Moreover, the test environments were different since we chose to test birds inside the isolators in order to avoid stress induced when removing them, while Diaz Heijtz et al. (2011); Crumeyrolle-Arias et al. (2014), and Neufeld et al. (2011) carried out behavioral tests outside the isolators. A third explanation would be that this modulation of emotional reactivity by gut microbiota would not always be due to a buffering effect. Stressful experiences have been shown to modify the composition of gut microbiota in rodents and horses (O'Mahony et al., 2009; Bailey et al., 2011; Mach et al., 2017) and this suggests a vicious circle situation where gut microbiota from emotional animals would be exposed to more host stress and would thus maintain, or even enhance, the stress state in the host. This explanation would be in line with recent data from our laboratory (data not shown) demonstrating that chronic mild stress protocol induces a marked change in gut microbiota composition in the quail line involved in this experiment. This would explain why emotional birds with microbiota would be even more emotional than germ-free emotional birds that would not experience this vicious circle effect.

### Effects on growth

Although studies on the influence of gut microbiota on behavior are rare in birds, work on its role in physiology and morphology is not recent (Gordon, 1959; Kussaibati et al., 1982; Mitsuhiro and Jun-ichi, 1994). In 1959, Gordon observed that the growth of specific pathogen-free chickens and germ-free chickens was not different. Several years later, Kussaibati et al. (1982) noted a slight increase in weight gain in germ-free chicks compared to specific pathogen-free chicks. In our study we found similar results since no significant difference was observed between GF and COL quails. It is an interesting result to discuss, because in rodents a significant difference in growth could be observed. Indeed, Gordon (1959) showed that growth was lower in germ-free rats and mice. More recently, Schwarzer et al. (2016) also noted that the growth of germ-free mice at 7 and 14 days of age was lower than that of specific pathogen-free mice. This contrast of results could be explained by the autonomous character of nidifugous birds that are not influenced by the feed provided by the mother, for example milk in mammals. It is important to note that this result allows us to confirm that the behavioral differences obtained in this study cannot be linked to a major disruption of growth.

## Conclusion

In conclusion, for the first time this study demonstrated that the absence of gut microbiota reduces emotional reactivity in Japanese quails. As in rodents and humans it is clear that there is an impact of gut microbiota on behavior in birds. However, information on the mechanisms and signaling pathways responsible for communication between microorganisms and the brain are still needed to support the existence of a microbiota-gut-brain axis in this model.

## Author contributions

CL, NK, MB, SR, EG, and PV conceived the study with the help of LC. NK, MB, and CL performed the experiments. NK and CL wrote the main part of the article. LC, SR, PV, MB, and EG reviewed the paper. All authors have read and approved the final manuscript.

### Conflict of interest statement

The authors declare that the research was conducted in the absence of any commercial or financial relationships that could be construed as a potential conflict of interest.
