# Supplementary material for: Absence of Gut Microbiota Reduces Emotional Reactivity in Japanese Quails (Coturnix japonica)
Source: Front Physiol. 2018 May 24;9:603. doi: 10.3389/fphys.2018.00603 (PMC5976779; doi:10.3389/fphys.2018.00603)
Supplement: Supplementary file 1 [file Table_1.docx]

Table S1: Statistical values for the effects of sex and its interaction with treatment in the behavioral tests. TI= Tonic Immobility test; SS= Social Separation test; NO= Novel Object test.

| Parameters | Treatment*Sex | | Sex | |
| --- | --- | --- | --- | --- |
|  | **χ^2^** | ***p*** | **χ^2^** | ***p*** |
| TI- Duration | 0.28 | 0.59 | 0.00 | 0.99 |
| TI- Number of inductions | 0.05 | 0.82 | 0.2 | 0.65 |
| TI- Index | 0.35 | 0.55 | 0.05 | 0.82 |
| SS- Total distance traveled | 0.68 | 0.41 | 2.73 | 0.12 |
| SS- Total velocity | 0.65 | 0.42 | 2.70 | 0.11 |
| SS- Number of entries in wall zone | 1.84 | 0.18 | 0.82 | 0.37 |
| SS- Entries in intermediate zone | 0.53 | 0.47 | 2.68 | 0.13 |
| SS- Entries in far zone | 1.79 | 0.18 | 2.82 | 0.11 |
| SS- Time spent in wall zone | 0.12 | 0.73 | 0.01 | 0.92 |
| SS- Time spent in intermediate zone | 0.30 | 0.58 | 0.03 | 0.86 |
| SS- Time spent in far zone | 1.17 | 0.28 | 0.24 | 0.63 |
| SS- Distance traveled in wall zone | 0.90 | 0.34 | 0.31 | 0.58 |
| SS- Velocity in wall zone | 1.41 | 0.23 | 2.14 | 0.15 |
| NO- Number of pecking object | 0.00 | 0.94 | 0.63 | 0.42 |
| NO- Number of moving object | 0.00 | 0.98 | 0.52 | 0.47 |
| NO- Time spent in wall zone | 0.01 | 0.94 | 0.20 | 0.90 |
| NO- Time spent in far object zone | 1.16 | 0.28 | 0.61 | 0.74 |
| NO- Time spent in moderate object zone | 1.76 | 0.21 | 1.10 | 0.58 |
| NO- Time spent in near object zone | 1.43 | 0.23 | 2.56 | 0.28 |
| NO- Time spent in object zone | 1.07 | 0.30 | 2.64 | 0.23 |
| NO- Time spent in near object zone (0-5 min) | 0.12 | 0.73 | 1.97 | 0.22 |
| NO- Time spent in near object zone (5-10 min) | 0.25 | 0.88 | 0.43 | 0.81 |
